# CD133^+ ^adult human retinal cells remain undifferentiated in Leukaemia Inhibitory Factor (LIF)

**DOI:** 10.1186/1471-2415-9-1

**Published:** 2009-02-23

**Authors:** Debra A Carter, Andrew D Dick, Eric J Mayer

**Affiliations:** 1 Academic Unit of Ophthalmology, Department of Clinical Sciences South Bristol, University of Bristol, Bristol Eye Hospital, Lower Maudlin Street, Bristol, BS1 2LX, UK

## Abstract

**Background:**

CD133 is a cell surface marker of haematopoietic stem and progenitor cells. Leukaemia inhibitory factor (LIF), sustains proliferation and not differentiation of embryonic stem cells. We used CD133 to purify adult human retinal cells and aimed to determine what effect LIF had on these cultures and whether they still had the ability to generate neurospheres.

**Methods:**

Retinal cell suspensions were derived from adult human post-mortem tissue with ethical approval. With magnetic automated cell sorting (MACS) CD133^+ ^retinal cells were enriched from post mortem adult human retina. CD133^+ ^retinal cell phenotype was analysed by flow cytometry and cultured cells were observed for proliferative capacity, neuropshere generation and differentiation with or without LIF supplementation.

**Results:**

We demonstrated purification (to 95%) of CD133^+ ^cells from adult human postmortem retina. Proliferating cells were identified through BrdU incorporation and expression of the proliferation markers Ki67 and Cyclin D1. CD133^+ ^retinal cells differentiated whilst forming neurospheres containing appropriate lineage markers including glia, neurons and photoreceptors. LIF maintained CD133^+ ^retinal cells in a proliferative and relatively undifferentiated state (Ki67, Cyclin D1 expression) without significant neurosphere generation. Differentiation whilst forming neurospheres was re-established on LIF withdrawal.

**Conclusion:**

These data support the evidence that CD133 expression characterises a population of cells within the resident adult human retina which have progenitor cell properties and that their turnover and differentiation is influenced by LIF. This may explain differences in retinal responses observed following disease or injury.

## Background

Neurospheres can be reliably generated from post-mortem adult human retina irrespective of post-mortem time (up to 48 hours) or age of donor [1-3]. This implies that the adult human retina may possess an inherent ability for cellular replacement throughout life. To this aim we have a presence of somatic progenitor cells, within adult human tissue, which unlike stem cell populations previously described for the retina, have limited potential but are responsible for cell renewal via differentiation into mature functioning cells. Progenitor cells divide rarely [4-6] but are likely to be stimulated to proliferate in the event of tissue damage due to disease or injury [5,7]. These progenitor cells are thought to differ from stem cells in that their proliferation is most likely asymmetric i.e. where some cells retain the properties of the parent cell and some begin the process of differentiation into mature cells [8]. The progenitor cells that we are studying within the retina are observed to have a reduced proliferation rate and generate neurospheres containing cells at different stages of differentiation [2]. This cannot be compared to the hallmarks of stem cells which have a high rate logarithmic expansion of totipotent cells usually associated with embryonic tissue but more recently identified within adult neural tissue [8]. Cell-cell, cell-matrix, cognate and soluble growth factor and cytokine-mediated regulation of cell may maintain retinal progenitor cells in an arrested or quiescent state with very low turnover [4-10]. In order to therapeutically support or facilitate progenitor cells roles in regeneration of adult retina following inflammation, degeneration or damage, further understanding of the control of cell turnover is required. The isolation or enrichment of this population of cells is therefore an important objective for this avenue of research. To date a few cell markers have proved suitable for specifically identifying, isolating or enriching progenitor cell populations [4,11]. A common marker for neural tissue progenitors is the cell surface expression of CD133 [4,12-15]. CD133 is a pentaspan glycoprotein expressed in the plasma membrane protrusions of cells, first identified on mouse neuroepithelial stem cells [16]. In humans CD133 identifies haemopoietic stem and progenitor cells that may also express the stem cell marker CD34 [17,18]. Although the function of CD133 in stem/progenitor cells is unknown, it is expressed in a wide range of tissues throughout the body [19]. Within the retina CD133, human prominin (mouse)-like 1 (PROML1, previously known as AC133 antigen) is concentrated in the membrane evaginations at the base of rod photoreceptor cell outer segments, (from where photoreceptor discs are formed) and in the absence of retinal prominin 1/CD133, photoreceptor degeneration occurs [20]. CD133 expression in undifferentiated cells has been utilised to identify, isolate and enrich stem cells [19,21] and neural progenitor from the developing and adult brain [4,12-15,22]. In these systems CD133^+ ^progenitor cells generated primary and secondary neurospheres (demonstrating potential for passage) [22]. Furthermore CD133^+ ^cell populations showed potent engraftment, proliferation, migration, and neural differentiation ability [14,15,22].

Following injury or damage, neural tissue responds with production of growth factor, neutrotrophins and cytokines including the mitogen, leukaemia inhibitory factor (LIF). LIF is a member of the IL-6 cytokine family that maintains stem cells in an undifferentiated state and promotes stem cell renewal [23]. In the adult rodent brain, LIF is localised to neurons within the olfactory sensory layer [24,25] and is upregulated in injury or tissue damage enhancing neural progenitor cell turnover [26]. LIF signals via gp130, (associated with LIF receptor) driving progenitor cells to re-enter the cell cycle [26,27]. Although LIF maintains neural stem cell turnover observed *in vivo*, exogenous LIF promotes gliogenesis alongside enhanced neurosphere production *in vitro *[27]. The actions of LIF may therefore vary in different contexts.

The data presented demonstrate successful purification of adult human progenitor cells through magnetic separation using the stem cell marker CD133. Purified cultures were assessed in the presence of growth factors and cytokines (FGF2 and LIF) for proliferative ability (markers of division), neurosphere generation and cell differentiation and phenotype. The role of exogenous LIF in maintaining retinal progenitor cells in culture (preventing neurosphere generation and differentiation) was also addressed. CD133^+^retinal cells showed increased proliferation in the presence of LIF as measured by BrdU incorporation, Ki67 and Cyclin D1 expression. CD133^+ ^retinal cells were efficient in forming neurospheres, which was suppressed in the presence of LIF and restored again by LIF removal, without any demonstrable change in their differentiation capacity within neurospheres.

## Methods

### Preparation of tissue

Research on human retinal tissue was carried out in compliance with the Helsinki Declaration. Donor tissue was obtained from Bristol Eye Bank after the removal of the cornea for transplant was used with research consent and ethical approval (Central and South Bristol Research and Ethics project number E5866) and R and D approval (United Bristol Healthcare project number OP/2004/1734). As previously reported, the retina was removed from donor eyes [1-3]. Post mortem times varied between 20 and 48 hours which does not alter the ability of progenitor cell cultures to generate neurospheres [3].

### Immunohistochemistry of retinal sections

Whole neural retina was removed from donor eyes and washed in cell culture medium to remove any contaminating retinal pigment epithelium (RPE) and then fixed in 1% paraformaldehyde for 2 hours at room temperature. After washing in PBS, whole neural retina was snap frozen in tissue tek (OCT) freezing matrix (Sakura, EU) in liquid nitrogen vapour. 3 × 12 μm sections were cut and captured on each poly-l-lysine coated slides. To stain, slides were immersed in PBS to rehydrate prior to adding the primary antibody (CD133, 1 μg/ml Miltenyi biotec, UK) for overnight incubation at 37°C and labelled with Alexa Fluor^® ^488 & 594 (Molecular Probes Inc, USA 1 μg/ml) secondary antibodies for 1 hour at room temperature. For dual staining, second specific mAb were added as above (Nestin, 2.5 μg/ml, R & D Systems, UK; Notch-1, 2.5 μg/ml, eBioscience, Europe and Pax-6, 4 μg/ml, Santa Cruz, UK). Actin was labelled on slides using rhodamine phalloidin (Molecular Probes Inc, USA 1 μg/ml) and counterstained with DAPI (Vector Shield Laboratories, UK). Slides were analysed with image capture using a Leitz (Dialux 22EB; Leica, Europe) fluorescent microscope.

### CD133 magnetic cell purification

**Figure 1:**
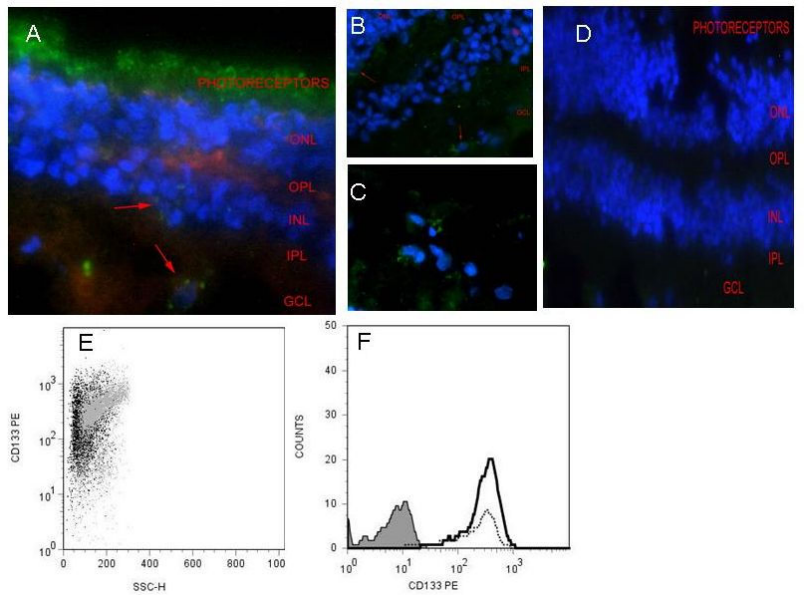
**CD133 expression within adult human retinal and following magnetic CD133 cell sorting**. (A) Frozen semi-thin retinal sections demonstrating CD133 expression within the photoreceptor layer (green) X400. Discrete CD133^+ ^cells are also identified within the inner nuclear layer (INL, red arrow A) and ganglion cell layer (GCL, red arrow A). Panel B concentrates on the OPL and GCL X400 and C represents magnification of the GCL X630 within adult human retina. red arrows identify CD133^+ ^cells (D) isotype control. Actin was labelled with rhodamine phalloidin and nuclei were counterstained with DAPI. Scale bars represent 0.5 μm (A, B, D) and 0.1 μm (C). Magnetic bead tagged -CD133 was used to isolate CD133^+ ^cells from primary retinal cell suspension via positive selection (E) CD133^+ ^expression was confirmed by flow cytometry in primary retinal cell culture (~67%) and is represented here as a FACS dot plot (grey) and following CD133^+ ^purification.(~96% purity) cells display varying extent of expression (black). (F) Histogram to show the increase in CD133^+ ^cells (black line) following magnetic separation from primary suspension (dotted line). The Grey filled peak represents isotype mAb fluorescence (0.82%).

Primary retinal cell suspensions were generated using standard methods [1-3]. Briefly, following exposure to enzymes (trypsin, collagenase, DNase) and triturated retinal cells were washed in buffer (0.5% FCS, 2 mM EDTA, PBS, Ph 7.5). After centrifugation the collected pellet was resuspended in 300 ml of ice cold buffer with 100 μl FcR block (Miltenyi Biotec, UK) and 100 μl magnetic CD133 (AC133 clone, recognising CD133/1 splice variant) beads (CD133 microbead kit, Miltenyi Biotec, UK), in accordance with manufacturer's instructions. Bead to cell ratios for maximal enrichment had been previously titrated to optimal levels 100 μl beads/1 × 10^8 ^cells. Cells were incubated at 4°C for 30 minutes. Cells conjugated to beads were then washed with buffer and applied onto a MS column, in 3 ml of buffer, of a MiniMACS CD133 separation kit (Miltenyi Biotec, UK). The CD133 depleted cells were collected as the effluent and the column again washed again 4 times in 3 ml of buffer to flush out positive cells. The CD133^+ ^cells collected were then passed down a fresh column to further purify the cell population (Fig 1A, B). Thus cell purification resulted on average a 95% CD133^+ ^cell population for all experiments.

### Flow cytometric cell surface phenotypic (FACS) analysis

Cell surface phenotype of CD133^+ ^retinal cells and CD133^- ^cells was assessed by multiparameter flow cytometry (FACS Calibur, BD biosciences). Monoclonal antibodies (mAb) to the following markers were used at previously titrated optimal dilutions and included: CD133 (clone AC141 293C3 [CD133/2 splice variant] recognising epitope 2 of huCD133), CD34, CD45, CD117 and CD271 (Miltenyi Biotec, UK). Doublecortin (DBX), Nestin, LIF receptor (LIFR), ABCG2, NCAM and matched isotype control mAb (R & D Systems, UK). Ki67, Cyclin D1 (ABCAM, UK)., CRALBP, CD90, GFAP, Vimentin, Rhodopsin and Pax-6 (Santa Cruz UK), Neurofilament M, Oligodendrocyte marker 4 (O4) (Chemicon, Europe), CD31, Recoverin, NANOG, Notch-1, CD135 (eBioscience, Europe), Glutamine Synthatase (BD transduction Labs, Europe). mAb were directly conjugated with combinations of APC, FITC or PE or unconjugated abs were labelled with the secondary antibodies alexa Fluor^® ^488 & 594 (Molecular Probes Europe). Intracellular antibodies were fixed and permeabilised with BD Cytofix/Cytoperm™ solution followed by washing with Perm/Wash buffer (BD Europe).

**Figure 2:**
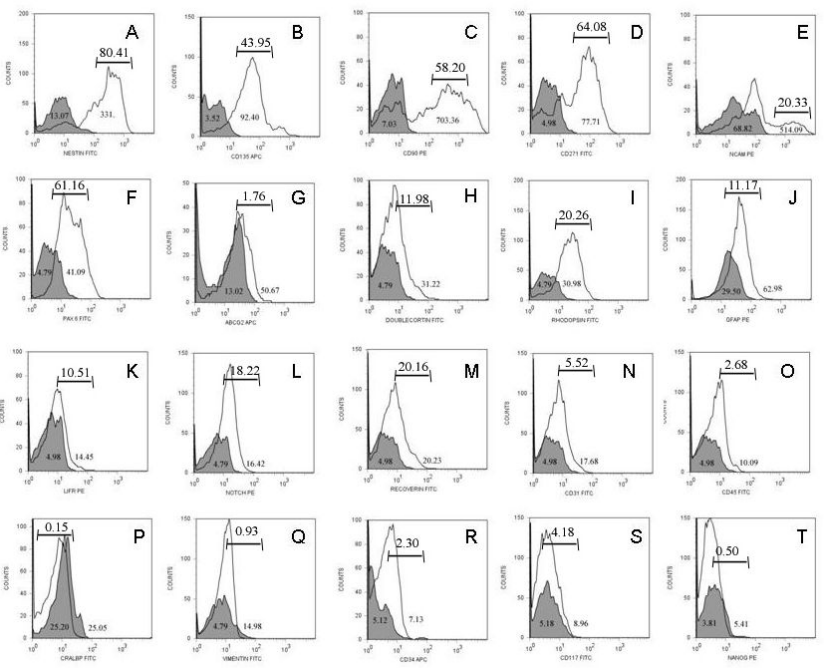
**flow cytometric analysis of CD133^+ ^retinal cells**. Fluorescent histograms showing relative expression of stem/progenitor cell markers NESTIN (A), CD135 (B), CD90 (C), CD271 (D), NCAM (E), Pax-6 (F), ABCG2 (G), DBX (H), RHOD (I), GFAP (J), LIFR (K), Notch-1 (L), RECOVERIN (M), CD31 (N), CD45 (O), CRALBP (P), VIMENTIN (Q), CD34 (R), cd117 (S) and NANOG (T) in CD133^+ ^sorted retinal cells. Grey filled peaks are isotype mAb background fluorescence for each specific mAb investigated. Numbers within peaks refer to mean fluorescent intensity (MFI), numbers above bars refer to positive percentage expression. The Correspondingly CD133^+^retinal cells have a phenotype of Nestin^hi^, CD135^hi^, CD90^hi^, CD271^hi^, NCAM^hi^, Pax-6^hi^, DBX^lo^, RHOD^lo^, GFAP^lo^, LIFR^lo^, Notch-1^lo^, RECOVERIN^lo ^and CD31^-^, CD45^-^, ABCG2^l-^, CRALBP^-^, VIMENTIN^-^, CD34^-^, CD117^-^, NANOG.

**Figure 3:**
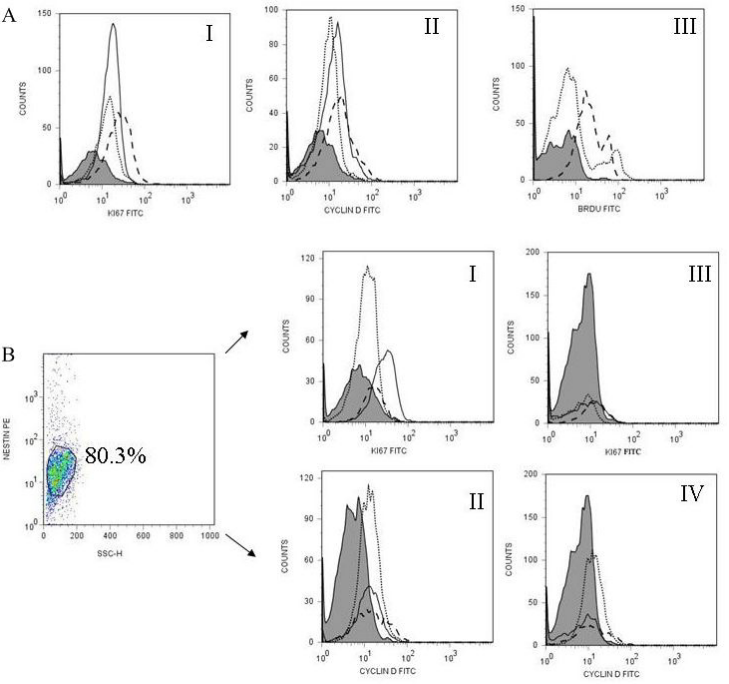
**CD133^+ ^retinal cells expression of proliferation markers following culture with FGF/N2 and FGF-LIF overnight**. CD133^+ ^retinal cells following culture overnight with either FGF/N2 or FGF-LIF were analysed by flow cytometry for the expression of the proliferation markers Ki67, Cyclin D1 or BrdU incorporation.(**A) **Histograms show the expression of Ki67 (AI) and Cyclin D1 (AII) after culture with FGF/N2 (dotted line) or FGF-LIF (dashed line) compared to expression immediately following CD133^+ ^retinal cell isolation (black line) against isotype control (Grey filled peaks). LIF within cultures causes an increase in expression of cell proliferation markers. Figure AIII shows incorporation of BrdU within cells following culture overnight with FGF/N2 (dotted line) or FGF-LIF (dashed line) indicating the generation of newly formed cells. **(B) **Dot plot demonstrates gated CD133^+ ^Nestin^+ ^events presented as mAb fluorescence against side scatter. CD133^+^Nestin^+ ^and Nestin^-^CD133^+ ^retinal cells were analysed for coexpression of Ki67 (I, III) or Cyclin D1 (II, IV). At time 0, Nestin positive cells expressed higher levels of proliferation markers Ki67 (I 5.90%, MFI 19.91) and Cyclin D 1 (II 12.01%, MFI.22.11) than nestin negative cells (Ki67 III Ki67 2.10%, MFI 11.19 and Cyclin D 1 IV 9.50%, MFI 19.07). Purified cells directly *ex vivo *(black line), culture overnight with FGF/N2 (dotted line) or FGF-LIF (dashed line).

**Figure 4:**
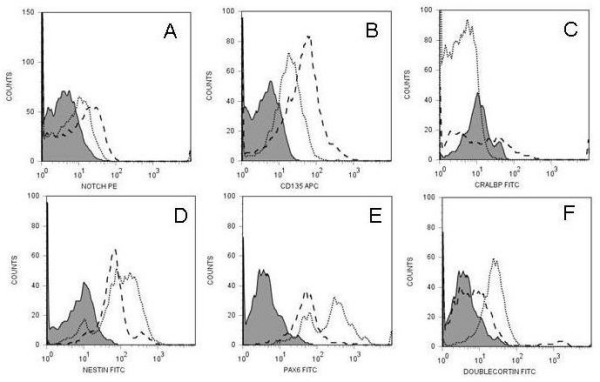
**phenotype of CD133^+ ^retinal cells following 24 hour culture**. The Figure demonstrates histograms of gated CD133 events showing relative expression of stem/progenitor cell markers that change when cells are cultured with either FGF/N2 or FGF-LIF overnight. Expression of Notch-1 (A), CD135 (B), CRALBP (C), Nestin (D), Pax-6 (E), DBX (F) changed on CD133^+ ^retinal cells following culture overnight with FGF/N2 (dotted line) or FGF-LIF (dashed line). Grey filled peaks are isotype mAb background fluorescence for cells cultured in FGF/N2 as MFI did not significantly change when cells were cultured in either culture condition.

BrdU was obtained from Pharmingen (Europe) and was visualised with anti BrdU following DNA denature (Chemicon, Europe). All flow cytometric analyses were conducted using FlowJo software (Tree Star, Inc. Oregon Corporation, USA). FACS data were interpreted as dot plots and histograms. Dot plots were generated of fluorescence mAb staining versus side scatter to display uniformity of cell population and display a horizontal line below which represent isotype mAb control background fluorescence levels. Histograms include percentage of positive events (number over gate) and levels of mean fluorescence intensity (MFI) for specific mAb cell surface expression (unfilled peaks) plus MFI for isotype control mAb (Grey filled peaks) (Figure 2, 3, 4).

### Affymetrix Analysis

300 ng of RNA were isolated from CD133^+ ^magnetically sorted cells using RNeasy mini^® ^kit (Qiagen, UK) following the manufacturers instructions. RNA was extracted from TIME 0 CD133^+ ^cells from 4 different eye donors. RNA quality was assessed using the Nanodrop1000 spectrophotometer (Labtech) and the 2100 Bioanalyser (Agilent Technologies). The University of Bristol Transcriptomics Facility carried out the analysis on Affymetrix GeneChip^® ^Human Gene 1.0 ST Arrays, using the Affymetrix whole transcript (W T) assay. Image analysis was carried out in Affymetrix GeneChip Command Console and probe set summarisation and preliminary data quality evaluation was carried out using Affymetrix Expression console software. Further analysis of the data including negative and positive controls was carried out using Affymetrix robust multiarray average (RMA). RMA values are multi-chip model-based relative expressions of arrays via background correction, log-transformation and normalization and gene-by-gene processing with robust median polish of *PM *values (perfect match) [28-30]. Microarray data discussed in this publication have been deposited within the NCBI's Gene Expression Omnibus (GEO Carter D A et al 2009) and are accessible through GEO Series accession number GSE14733 .

### Cell Culture

Following magnetic purification, CD133^+ ^retinal cells were seeded into T25 flasks at 1 × 10^6 ^cells/ml. Primary retinal cells and run through CD133 depleted cells were cultured along side CD133^+ ^retinal cells as positive and negative controls. Cells were cultured in DMEM/nutrient mix F12 with glutamax-1 (L-Alanyl-L-Glutamine 1:1) with pyridoxine (DMEM/F12 GIBCO, UK) which was supplemented with 20 ng/ml FGF2 (SIGMA, UK) and either neural supplement (N2) (GIBCO, UK) with or without 100 ng/ml LIF (Chemicon, UK). Optimum culture conditions were medium containing both FGF2 and N2 (FGF2/N2) [2,3]. For LIF removal studies, cells were initially cultured for 3 days within FGF2/N2 supplemented with LIF (FGF2/N2-LIF). As LIF has been found to exert its effects within 72 hours of exposure [30], LIF was removed from the medium following 3 days in culture by spinning cells and reseeding them into FGF2/N2 medium with subsequent culture to generate neurospheres.

### Neurosphere Scoring (pre staining)

Neurospheres were counted within flasks following 1–3 weeks in culture. Neurospheres were identified as free floating balls of cells that were phase bright. Cell aggregates were distinguished by having a dark centre when observed with a phase contrast microscope and in addition tended not to be free floating in suspension rather adherent to the flask.

### Immunofluorescence studies

Neurospheres were fixed in 1% paraformaldehyde for 20 minutes at room temperature and then washed with PBS (spinning for 7 mins @ 2500 rpm × 2). Human FcR block (Miltenyi Biotec, UK) and diluted Primary antibodies were added and incubated overnight at 4°C. Antibodies used included: GFAP (Chemicon USA 1 μg/ml), Neurofilament M (NF, Chemicon, USA 5 μg/ml), Nestin (Chemicon, US 10 μg/ml) and doublecortin (DBX, R & D 1 ng/ml). After washing with PBS, cells were incubated with Alexa Fluor^® ^488 & 594 (Molecular Probes Inc, USA 1 μg/ml) secondary antibodies for 1 hour at room temperature. For dual staining, second specific mAb were added as above. Neurospheres were finally air dried onto a slide and nuclei were counterstained with DAPI. Slides were viewed and marker expression analysed using a Leitz (Dialux 22EB; Leica, Europe) fluorescent microscope at ×40 magnification. Images were taken using a Leica (Europe) AOBS SP2 confocal imaging system attached to a Leica DM IRE2 inverted epifluorescence microscope.

### Neurosphere Scoring (post staining)

Following staining, neurospheres were identified as dapi postitive balls of cells containing more then 4 cells. Within each neurosphere the number of dapi positive cells was counted and positive staining of markers was noted for each cell. Ten fields of view were counted for each slide and approximately 5 slides from different experiments were assessed.

### ELISA

Determining LIF concentrations within culture supernatants was assessed using human LIF Quantikine^® ^(R & D systems, UK) as per manufacturer's instructions. In brief, duplicate of samples and LIF standards (serially diluted in phosphate buffer) were added to 96 well plate pre-coated with mouse-anti human LIF mAb. The plate was incubated at room temperature for 1 hour. A Wash buffer (containing 0.05%Tween-20) was used between steps. LIF conjugate was added to each well and incubated for 1 hour at room temperature. Substrate solution was added for 30 minutes then within 30 minutes of stopping the reaction the absorbance was read at 450 nm corrected to 570 nm using a SpectraMax spectrophotometer (Molecular Devices, USA), pre-blanking with assay diluent. SoftMax Pro software (Molecular Devices, USA) was used for analysis and transformation to generate a standard curve and cytokine concentration was then determined using computer software Excel (Microsoft^®^). Each ELISA was repeated at least three times in independent experiments and results expressed as a mean. ± SEM.

### Statistical Analysis

Analysis was performed using StatView^® ^from the SAS Institute Inc (USA). Non parametric tests were used to analyse all data. Neurosphere generation was first corrected to neurospheres per 100000 live cells (NS/100000). Mann Whitney testing was used to look at 2 group data and Kruskall-Wallis tests were used to look at 3 group data.

## Results

### Isolation of CD133^+ ^retinal cells from post-mortem human retina

In post mortem adult human, CD133^+ ^expression is distributed within the photoreceptor layer of retina (Figure 1A) as well as discrete cells within the inner nuclear layer (INL) and ganglion cell layer (GCL) (Figure 1B–C). FACS analysis of retinal cell suspensions, demonstrated CD133 expression on approximately 67% of cells, including prominin-1 expressing photoreceptor cells [21,22]. Magnetic separation using CD133-conjugated beads allowed purification of the primary retinal cell suspension (Figure 1E–F). The CD133^+ ^retinal cells, (initially including CD133^+ ^photoreceptors), were purified by 31.2 ± 2.4% (n = 10). Starting with average of 5 × 10^6 ^primary retinal cells, post CD133-isolation a purity of 95% CD133^+ ^retinal cells was achieved. FACS dot plots showed that post-sort a change in the cell scatter (Figure 1E) and increase in cell counts (Figure 1F).

### Phenotype of CD133^+ ^retinal cells directly ex vivo and following culture supplemented with LIF

Initially we characterised the phenotype of CD133^+ ^and CD133^- ^cell populations following magnetic sorting at time 0 in culture by flow cytometry. CD133^- ^cell populations showed no significant expression of stem cell markers but expressed high levels of differentiated cell markers including photoreceptors (rhodopsin), neurons (neurofilament), ganglion cells (CD90), Muller glia cells (CRALBP) and glial cells (GFAP) plus low levels of other heamopoeitic cell markers (CD45, CD31 and CD34) (see additional file 1). CD133^+ ^retinal cells showed relative expression of stem/progenitor cell markers (Figure 2) [31-35]. CD133^+ ^retinal cells expressed high levels of Nestin (Figure 2A, 80.14%, MFI 331, isotype control 13.07), CD135 (Figure 2B, 43.95%, MF1 92.40, isotype control 3.52), CD90 (Figure 2C, 58.20%, MFI 703.36 isotype control 7.03), CD271, (Figure 2D, 64.08, MFI 77.71, isotype control 4.98), NCAM, (Figure 2E, 20.23%, MFI 514.09, isotype control 68.82), Pax-6 (Figure 2F, 61.16, MFI 41.09, isotype control 4.79), and low levels of DBX (Figure 2H, 11.98%, MFI 31.22, isotype control 4.79), GFAP (Figure 2J, 11.17%, MFI 62.98, isotype control 29.50), Rhodopsin (Figure 2I, \20.26%, MFI 30.98, isotype control 4.79), LIFR (Figure 2K, 10.51%, MFI 14.45, isotype control 4.98), Notch-1 (Figure 2L, 18.22%, MFI 16.42, isotype control 4.79), Recoverin (Figure 2M, 20.16%, MFI 20.23, isotype control 4.98). The CD133^+ ^retinal cells did not express significant levels of CD31 (Figure 2N, 5.52% MFI 17.68, isotype control 4.98), CD45 (Figure 2N, 2.68%, MFI 10.09, isotype control 4.98) and ABCG2 (Figure 2G, 1.76% MFI 50.67, isotype control 13.02), and were negative for CRALBP (Figure 2P, 0.15%, MFI 25.05, isotype control 25.20), VIMENTIN (Figure 2Q, 0.93%, MFI 14.98, isotype control 4.79), CD34 (Figure 2R, 2.30%, MFI 7.13, isotype control 5.12), CD117 (Figure 2S, 4.18%, MFI 8.96, isotype control 5.18) and, NANOG (Figure 2T, 0.5%, MFI 5.41, isotype control 3.81) plus O4 (0.2%), NF-M (0.16%) and Glutamine synthatase (0.05%) (results not shown). In summary CD133^+ ^retinal cells purified from adult human retina have a phenotype of Nestin^hi^, CD135^hi^, CD90^hi^, CD271^hi^, NCAM^hi^, Pax-6^hi^,, DBX^lo^, Rhodopsin^lo^, GFAP^lo^, LIFR^lo^, Notch-1^lo^, RECOVERIN^lo^, and CD31^-^, CD45^- ^ABCG2^-^, CRALBP^-^, VIMENTIN^-^, CD34^-^, CD117^-^, NANOG^-^, O4^-^, NF-M^-^, Glutamine Synthatase^-^. The expression of the proliferation markers were assessed on CD133^+ ^retinal cells (Figure 3). Low level expression of Ki67, 27.37%, MFI 19.99, isotype control 4.98 (Figure 3A, I) and Cyclin D1, 15.83%, MFI 16.89, isotype control 4.98 (Figure 3AII) was found following isolation from the tissue (Time 0). Figure 3B displays Nestin^+ ^CD133^+ ^(Figure 3B, I & II) and Nestin^-^CD133^+ ^(Figure 3B III & IV). Nestin^+ ^CD133^+ ^(Figure 3B I – II) retinal cells are also positive for Ki67 (5.90%, MFI 19.91) and Cyclin D1 (12.01%, MFI.22.11), isotype control (1.31% MFI 4.98). Nestin^-^CD133^+ ^retinal cells expressed reduced levels of proliferation markers (Ki67 2.10%, MFI 11.19, Cyclin D 9.50%, MFI 19.07. Confirmatory transcriptonomic analysis supported the gene expression profile of CD133^+ ^cells; displaying correspondence between detection of mRNA expression and protein expression determined by FACS analysis, including the proliferation markers Ki67 and Cyclin D1 (table 1) [28-30].

**Table 1:** expression data for CD133^+ ^retinal progenitor cell phenotype

**mRNA Description**	**Mean Expression**
Homo sapiens prominin 1 (PROM1), mRNA.	6795

Homo sapiens nestin (NES), mRNA.	215

Homo sapiens fms-related tyrosine kinase 3 (FLT3), mRNA. (CD135)	66

Homo sapiens Thy-1 cell surface antigen (THY1), mRNA. (CD90)	390

Homo sapiens nerve growth factor receptor (TNFR superfamily, member 16) (NGFR), mRNA. (CD271)	542

Homo sapiens neural cell adhesion molecule 1 (NCAM1), transcript variant 2, mRNA.	1987

Homo sapiens paired box 6 (PAX6), transcript variant 1, mRNA.	788

Homo sapiens ATP-binding cassette, sub-family G (WHITE), member 2 (ABCG2), mRNA.	146

Homo sapiens doublecortex; lissencephaly, X-linked (doublecortin) (DCX), transcript variant 1, mRNA.	258

Homo sapiens rhodopsin (RHO), mRNA.	16078

Homo sapiens glial fibrillary acidic protein (GFAP), mRNA.	637

Homo sapiens leukemia inhibitory factor receptor alpha (LIFR), mRNA.	1737

Homo sapiens Notch homolog 1, translocation-associated (Drosophila) (NOTCH1), mRNA.	326

Homo sapiens recoverin (RCVRN), mRNA.	10323

Homo sapiens platelet/endothelial cell adhesion molecule (CD31 antigen) (PECAM1), mRNA.	188

Homo sapiens protein tyrosine phosphatase, receptor type, C (PTPRC), transcript variant 1, mRNA. (CD45)	38

Homo sapiens retinaldehyde binding protein 1-like 1 (RLBP1L1), mRNA (CRALBP1)	256

Homo sapiens vimentin (VIM), mRNA.	2709

Homo sapiens CD34 molecule (CD34), transcript variant 2, mRNA.	123

Homo sapiens KIT ligand (KITLG), transcript variant b, mRNA. (CD117)	136

Homo sapiens Nanog homeobox (NANOG), mRNA.	111

Homo sapiens neurofilament, medium polypeptide 150 kDa (NEFM), transcript variant 1, mRNA.	662

Homo sapiens cyclin D1 (CCND1), mRNA.	369

Homo sapiens antigen identified by monoclonal antibody Ki-67 (MKI67), mRNA.	55

RNA was isolated from magnetically separated CD133^+ ^cells and prepared using the Affymetrix whole transcript (WT) assay. Mean expression values represent 3 repeats of actively transcribing genes (GEO series record GSE14733). Although expressivity has varying values with respect to extent of protein expression via FACS analysis, we do not make comparisons at this level. Levels of mRNA are not directly proportional to the expression level of the proteins [30]. The number of protein molecules synthesized using a given mRNA molecule as a template is highly dependent on translation-initiation features of the mRNA sequence. In addition proteins may differ substantially in their *in vivo *half lives [30].

### Change of CD133^+ ^retinal cell phenotype following overnight culture with FGF/N2 or FGFN2-LIF

Figure 4 displays flow cytometric histograms following 24 hour culture in the presence of FGF2/N2 and with LIF supplementation. Following culture with FGF/N2-LIF for one day CD133^+ ^retinal cells which alters their expression of Nestin, CRALBP, DBX, Notch-1, Pax-6 and CD135 (Figure 4A–F). The effect of LIF on CD133^+ ^retinal cells causes a more pronounced increase in expression of Notch-1, CD135 and the Müller glia cell marker CRALBP (Figure 4 A–C). Conversely, culture overnight with FGF/N2 causes a more pronounced increase in Nestin, DBX and Pax-6 (Figure 4 D–F). Proliferation markers were also upregulated following incubation with LIF overnight Ki67, 51.54%, MFI 37.98, isotype control 4.98 (Figure 3AI) and Cyclin D1, 10.02%, MFI 24.83, isotype control (Figure 3AII). In addition FACS analysis revealed BrdU incorporation in CD133^+ ^retinal cells cultured with LIF overnight (7.16%, MFI 26.32, isotype control 4.98 Figure 3A III) overnight.

### FGF2/N2 Optimises Generation of Neurospheres from CD133^+ ^Cell Suspensions

**Figure 5:**
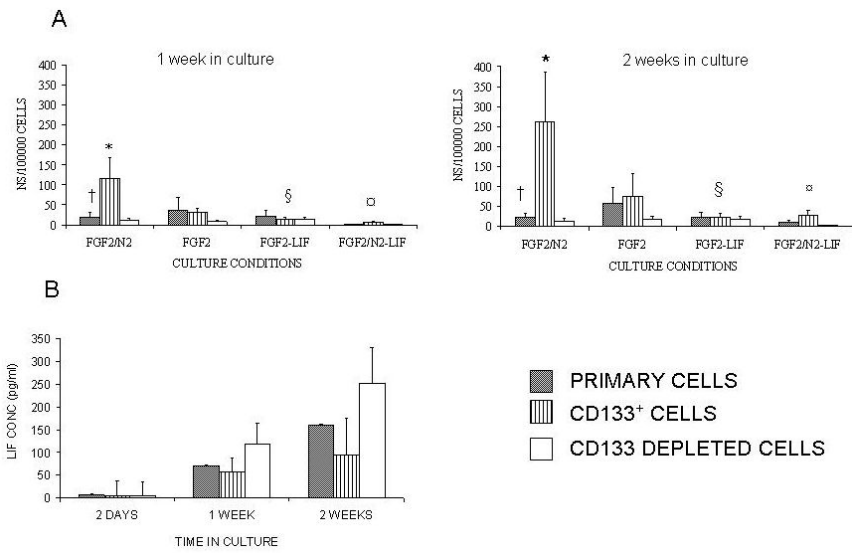
**FGF2/N2 optimises generation of neurospheres from CD133^+ ^retinal cells**. Primary, CD133^+ ^and CD133 depleted cells were cultured for 1 week, post-sort, with FGF2, FGF2/N2, FGF2-LIF and FGF2/N2 -LIF. Non parametric statistical analysis for neurosphere generation at 1 week **(A) **Neurosphere generation was enhanced when cells were cultured in FGF2/N2: At 1 week † Primary:CD133^+^:depleted P < 0.02, * Primary:CD133^+ ^P < 0.02 (n = 14). At 2 weeks: † Primary:CD133^+^:depleted P < 0.01, *Primary:CD133^+ ^P < 0.03 (n = 10). Neurosphere generation/100000 was significantly reduced in the presence of LIF (§ ¤ P < 0.05).**(B) **Supernatants from CD133^+ ^cultures were removed following 2 days, 1 week and 2 weeks and analysed for LIF production (pg/ml). LIF (pg/ml) increased over 2 weeks. However it was noted that CD133^+ ^retinal cells contained less LIF (pg/ml) over 2 weeks in culture which corresponded to increased neurosphere generation.

Given the evidence that in short-term culture, LIF supplementation maintained expression of progenitor cell markers, we assessed whether LIF maintained retinal progenitor cells and prevented neurosphere generation and differentiation. To do this CD133^+ ^and CD133- depleted cell suspensions were cultured in FGF2/N2 with or without LIF were compared for neurosphere generation (Figure 5). Neurosphere generation (expressed as the number generated per 100000 cells) was greatest in the presence of FGF2/N2, as previously documented [3]. FGF2/N2 culture supported optimum conditions for neurosphere generation. Additionally we found that CD133^+ ^retinal cells in FGF2/N2 culture generated significantly more neurospheres than primary cell suspensions after 1 week (117 ± 52 compared with 20 ± 10, P < 0.02 this was maintained at 2 weeks (261 ± 124 compared with 22 ± 11, P < 0.003) (Figure 5A). Analysis of LIF concentrations found them to be correspondingly reduced in CD133^+ ^cell FGF2/N2 (Figure 5B). At 2 days in culture, all culture supernatants (primary, CD133^+ ^and CD133-depleted) contained barely detectable quantities of LIF (< 10 pg/ml), but over time LIF production increased significantly, although remaining lower in CD133^+ ^retinal cells, corresponding to their increased ability to generate neurospheres. The ability of CD133^+ ^retinal cells to generate neurospheres was significantly suppressed in the presence of exogenous LIF at greater concentrations than those ever generated in culture (100 ng/ml vs. 100 pg/ml respectively) (following 2 weeks FGF2-LIF 23 ± 9, FGF2/N2-LIF 28 ± 11 P < 0.05) (Figure 5A).

### LIF withdrawal does not restore Neurosphere Generation to optimum levels

**Figure 6:**
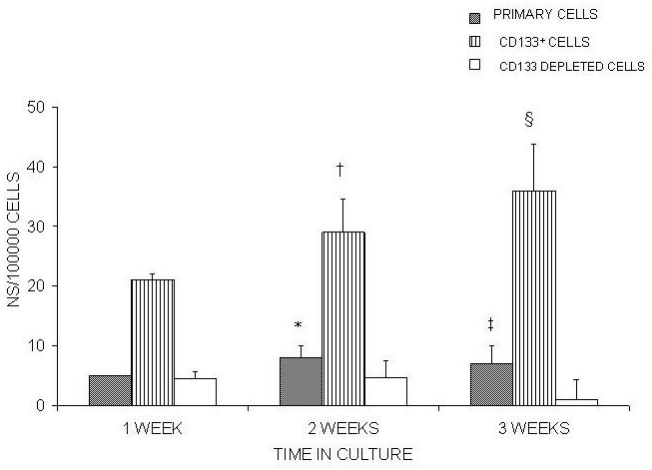
**withdrawal of LIF does not recover neurosphere generation in CD133^+ ^retinal cells to optimum levels**. Following the observation that LIF in the culture medium prevented CD133^+ ^retinal cells from generating neurospheres, LIF was removed after 3 days (see methods) by spinning down the cells and the medium was replaced with FGF2/N2 which was found previously to have a significant effect on neurosphere generation (Fig 4). After 1 week in culture with FGF2/N2 neurosphere generation increased significantly – †Primary P < 0.02 (n = 10) * CD133^+ ^P < 0.03 (n = 10). After 2 weeks in culture CD133^+ ^retinal cells were found to show a significant difference in neurosphere generation/100000 cells at 1 week §P < 0.02. CD133^+ ^retinal cells generated a significant number of neurospheres/100000 cells when compared to primary cultures at 2 weeks ‡ P < 0.04.

To assess whether the above mentioned LIF suppression of neurosphere generation was reversible, after 3 days in culture with FGF2/N2-LIF, LIF was removed by spinning cells and replacing medium with that for optimum neurosphere generation, FGF2/N2 (Figure 6). Within 7 days neurosphere generation by CD133^+ ^retinal cells, that had initially been exposed to LIF, recovered significantly (P < 0.05) and this remained true at 2 weeks (P < 0.03, Fig 6). CD133^+ ^retinal cells generated significantly more neurospheres/100000 cells when compared to primary cell culture at 2 weeks following LIF removal (P < 0.04, Fig 6) however neurosphere generation did not recover to optimum levels found when culturing cells with FGF/N2.

### Neurospheres Express Retinal Cell and Progenitor Cell Markers

**Figure 7:**
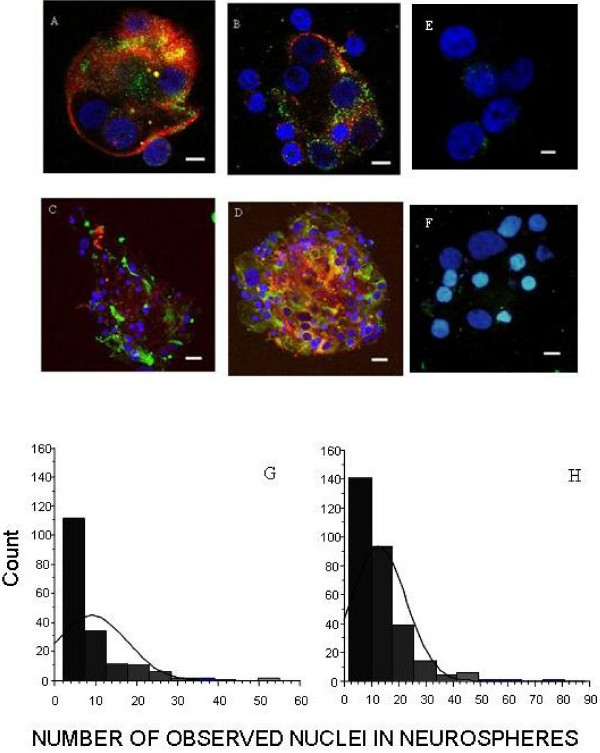
**neurospheres express retinal cell and stem cell markers**. Confocal Images of Neurospheres Generated from CD133^+ ^retinal cells cultured in FGF2/N2 **A **Neurosphere expressing Nestin^+ ^(red) and DBX^+^cells (green X630) **B **Neurosphere expressing NF^+ ^(red) and DBX^+ ^cells (green) X630. **C **Neurosphere expressing Nestin^+^(red) and GFAP^+ ^cells (green X400). **D **Neurosphere expressing Nestin^+ ^(red) and RHOD^+ ^cells (green X400). Nuclei counterstained with DAPI (blue). Neurospheres stained with isotype control antibodies did not have any significant fluorescent staining (E, X630, F, X630). Scale bars represent 5 μm (A, B, E) and 10 μm (C, D, F). Histograms represent the distribution of nuclei within neurospheres that have been generated within (G) FGF2/N2 and (H) FGF2/N2 following LIF removal (see methods). Neurospheres generated with pre exposure to LIF had significantly more nuclei within each neurosphere (P < 0.05).

After 2 weeks in culture neurospheres cultured in FGF2/N2 were compared with neurospheres cultured initially in FGF2/N2-LIF followed by LIF removal (see above). The neurospheres were stained for cell-type specific markers (GFAP, NF and Rhodopsin) to identify differentiated cells of glial, neuronal and photoreceptor cell lineage, as well as Nestin and DBX to identify less differentiated cells (Figure 7). Within each neurosphere, the number of nuclei were counted and each nucleus was assessed for positive staining as above. This quantification of both dual and single stained cells within each neurosphere allowed for both cell counts as well as semi-quantification of differentiation within neurospheres. Neurospheres generated within FGF2/N2 and FGF2/N2 following LIF removal contained cells of different lineages and did not contain significantly different mean numbers of glial, neurons and photoreceptors (FGF2/N2 36.6 ± 10.2, FGF2/N2 following LIF removal 30.5 ± 10.2) (Figure 7A–D). Neurospheres were found to contain cells with the developmentally regulated cytoskeletal proteins Nestin and/or DBX, both markers of multi-lineage progenitor cells [35-38]. At 3 weeks in culture, nuclear counts per neurosphere were on average 9 ± 1 in CD133^+ ^cell cultures in FGF2/N2. 13 ± 1 in FGF2/N2 when previously supplemented with LIF (Figure 7G, H; p < 0.0001). Initial exposure to LIF generated significantly larger neurospheres without influencing the differentiated cell types present within the sphere, although the overall number of neurospheres remained lower.

## Discussion

We have shown previously that the adult human retina contains potential progenitor cells, which although an infrequent cell type and likely to be distinct or potentially derived from stem cell niches described in ciliary body [39], can be cultured regardless of donor age or post mortem time – up to 48 hours [3]. Building on previous research showing that the retina has potential for repair and/or regeneration through the presence of specialised cells [1-3,10,39,40], we have isolated a population of CD133^+ ^cells from neural adult human retinal which possess a phenotype and properties that taken together is highly suggestive of a progenitor cell status. Cohnheim in 1867 coined the concept that stem cells originate within the blood stream and have the potential to exist in different tissues where their proliferative and differentiation ability is dependent upon the environment which they are exposed to [8]. Thus these progenitor cells may remain quiescent and turnover tissue at a rate that is unobserved due to the restraints of the microenvironment such as seen within the retina [4-8]. Within the retina, CD133^+ ^cells are subject to a complex microenvironment, which is likely to act through cytokines such as LIF and IL-6 and undoubtedly other mitogens that may regulate the mitotic activity and differentitation of these cells into mature cell types. Through the incorporation of BrdU we have shown that CD133^+ ^retinal cells do give rise to daughter cells that have low level proliferative capacity forming neurospheres that contain subset of cells that retain multipotency, expressing retinal cell markers including glial, neurons and photoreceptors.

Although our research focuses on CD133^+ ^cells within the retina, the exact origin of these cells remains an open question. Retinal progenitor cells may be derived from marrow progenitors, via the circulation (through retinal vessels, choroidal vessels and crossing Bruch's membrane or anterior eye structures); arise from a tissue specific population within the eye (or optic nerve) or arise from changes in other cells e.g. Müller glial or endothelial progenitor cells. The network of elongated Nestin^+ ^cells within the retina would support the distribution of migrating progenitors [1] but does not in itself help with the understanding of the origin of retinal progenitors, unless these cells themselves are the progenitors. Retinal stem cells migrate much more widely through the eye if injected into the vitreous than if injected under the retina however transplanted neural progenitor cells exhibit widespread migration into diseased retina [42-44]. Other groups have identified progenitor cells within the ciliary body [39,40],, [45-47], peripheral margin of the postnatal retina [45,46], iris pigment epithelium [50-52] and some suggest that Müller glial cells within the adult retina possess progenitor like properties [53,54]. We did not detect any markers on CD133^+ ^retinal cells to suggest that they were Müller Glial cells. Their lack of significant expression of haemopoeitic stem cell markers (CD34, CD117, ABCG2, CD45) may suggest that these cells are unlikely to have migrated from bone marrow [55-57].

The use of CD133 in purifying progenitor cells from both embryonic (murine and human) and adult brain (murine) is established and recognised [15,22]. The current data demonstrate that the same is true for human retinal progenitor cells. Primary cell suspensions from adult human retina generate more neurospheres per viable cells after selecting for CD133^+ ^retinal cells. This suggests that retinal progenitors may have similarity to those from embryonic brain [4,58]. The data presented demonstrate that a population of CD133^+ ^retinal cells have a greater capacity to generate neurospheres (a behaviour associated with differentiation of cells). Correspondingly FACS analysis revealed BrdU incorporation within CD133^+ ^retinal cells when incubated with FGF/N2, supporting the hypothesis that increases in overall cell numbers in cultures arise from newly generated cells.

The analysis of markers expressed by CD133^+ ^enriched retinal cells is consistent with a progenitor cell phenotype and is similar to other progenitor cells within the central nervous system [15,43,58]. As expected CD133^+ ^retinal cells highly express Nestin, a neural progenitor cell marker expressed within human retina [1], but in addition, there was a high expression of CD90, the neural receptor CD271, the neural adhesion marker NCAM, PAX-6 and the FIk2/Fit3 tyrosine kinase receptor CD135. PAX-6 is an important marker as it has a multifunctional role which includes regulating proliferation and differentiation through controlling expression of downstream molecules which contributes to the multipotency of retinal progenitor cells. Pax-6 influences the expression of the following transcription factors which ultimately leads to the generation of specific cell types; Math5, ganglion cells; NgN2, bipolar cells; Neuro D, amacrine cells; Crx1, photoreceptors; Rx1, muller glial cells and Pax-6 acts directly for horizontal cells and is essential for lens formation [59,60]. Previous studies have shown that Pax-6 is also important for neurosphere generation [61]. In these studies the Pax-6 gene deletion led to arrested retinal development and when cells from the optic nerve of these embryos were harvested, retinal neurospheres failed to develop [61]. This generates a significant role of Pax-6 in the competence of retinal stem cells and not just retinal development [61]. We provide the first description of retinal expression of CD135 on CD133^+ ^retinal cells, which has previously only be found to be expressed in fetal and adult brain, testis and the placenta. It is expressed in populations enriched for stem cells and primitive uncommitted progenitors including haemopoietic stem cells [62-64] and is currently being used as a tool for expanding stem cell populations [64]. The low and absent expression of the cells markers rhodopsin, GFAP, CD45, CD31, CRALBP and CD31 confirm that CD133^+ ^retinal cells alongside proliferation markers, are at least as subgroups not differentiated photoreceptors, glial, endothelial or immune cells.

The CD133 enrichment of adult human retinal cells convincingly enriches retinal neural progenitor cells. In support of this we noted that CD133^+ ^retinal cells upregulate their expression of Notch-1 when incubated with LIF which suggests that LIF would be expected to preserve undifferentiated progenitor cells in this system. An upregulation of Notch-1 is critical in the signalling pathway that preserves a pool of undifferentiated progenitor cells [65,66]. Low or absent levels of Notch-1 permit cells to exit the cell cycle and ultimately differentiate. [65,66]. Furthermore increased Notch-1 expression was accompanied by an upregulation of CD135 which as previously discussed, is also important for stem cell turnover. It is therefore not surprising that the cell cycle proliferation markers Ki67 and Cyclin D1 are also upregulated following LIF incubation. Cyclin D1 is more closely associated with promoting photoreceptor development [67]. Conversely both Pax-6 and Nestin are upregulated following incubation with FGF/N2, which seems to support the development of neurospheres and differentiated cells in our culture systems. These findings are consistent with current research on progenitor cell differentiation [36,61,68,69] As Pax-6 levels increase, progenitor cells leave the cell cycle and reach a pre-differentiated state before switching off and terminally differentiating [62,69]. Low levels of Pax-6 function to maintain progenitor cells within the cell cycle [61] which is in agreement with the lower expression found on CD133^+ ^retinal cells at time 0 and following LIF incubation. The increased expression of Nestin in FGF/N2 is not that surprising as this is where we see optimal neurosphere generation. The intermediate filament, Nestin has a structure which allows conformational change and as cellular differentiation is associated with morphological changes, following exposure to extracellular as well as cellular signals (such as those associated with the cell cycle), Nestin responds accordingly [36,69]. In our results we also saw an upregulation of DBX following incubation with FGF/N2. DBX is a microtubule protein which is generally expressed in migrating neuronal processes and more recently has been identified as a marker for neurogenesis [70]. Within our studies DBX expression could represent the presence of neuron generation and add more scope to the identity of CD133^+ ^retinal cells as multipotential progenitors.

In the central nervous system LIF is involved in responses to injury where expression of LIF increases following brain injury [26] with enhanced cell turnover and ensuing cell death and replacement in the damaged area [26]. Studies on LIF-mediated control of embryonic stem cells have suggested that self-renewal of cells involves an auto-regulatory loop [31]. In low concentrations of LIF, embryonic stem cells (ESCs) differentiate [31]. ESCs were found to commit to cell differentiation within the first 36 hours of LIF deprivation [31,71]. Low levels of LIF maintain cells in a non-responsive state due to endogenous STAT3 activity and once cells have lost STAT3 activity; LIF responsiveness is also lost [31]. Thus cells that have lost LIF-responsiveness are committed to differentiation [31], and this would reflect the findings in our culture system as when LIF is not added exogenously neurospheres are generated. In addition the lack of NANOG expression within our CD133^+ ^retinal cells suggests the need for LIF in order to self renew [72]. NANOG alleviates the necessity for LIF in ESC [72]. We found that CD133^+ ^retinal cells respond to LIF and respond in the manner expected of ESCs and progenitors described previously [27,31,73-75]. So when CD133^+ ^retinal cells were cultured in the presence of exogenous LIF; neurosphere generation was reduced. The suppression of neurosphere formation in the presence of LIF raises the question as to whether, like other neural progenitor cells, it may be possible to expand isolated retinal progenitor cells *ex vivo*. A question we hope to address fully in future work.

Despite our observed effect of exogenous LIF on CD133^+ ^retinal cells, neurospheres generated within the optimum growth condition FGF/N2 occurred in the presence of low levels of endogenous LIF. The low levels of LIF at 48 hours increased to significant levels at 2 weeks. For ESCs, LIF concentration is converted into an all or nothing response [31]. Applying this mechanism to CD133^+ ^retinal cells the level of endogenously generated LIF may initially have been below the threshold to change the fate of CD133^+ ^retinal cells, thus neurosphere generation ensued. Neurosphere differentiation could not be reversed when LIF was re-introduced (unpublished observations) [31] and we do not have any evidence as to which cells generate LIF in our cultures.

Studies of embryonic neural precursor cells found that prior exposure to LIF enhances the generation of neurospheres in the presence of appropriate growth factors [31]. Ability to generate neurospheres from purified CD133^+ ^adult human retinal cell suspensions was regained after LIF removal but did not fully recover to the extent seen in cells never exposed to LIF, where neurospheres were generated from CD133^+ ^cells in FGF/N2 alone. The reason remains unknown but may relate to the rate of cell turnover (which is low for adult human-derived cells). Different stem/progenitor cells have different rates of turnover [4-8,75]; faster turnover is observed with embryonic tissue and tissue from small species, where there is a high index of cell division and cell death in differentiated cells [8,75]. Conversely stem cells that turnover slowly and thus divide infrequently generate long lived differentiated cells; a trait arguably favourable within adult tissues [8,77]. Pre-exposure to LIF did influence the size of neurospheres generated; so that they contained significantly more nuclei than neurospheres simply cultured in FGF2/N2. This was also found when neurospheres were generated from neural progenitor cells of postnatal rats that were exposed to LIF [76]. So although the reason for this is unclear it is not a unique observation. Autoregulation of LIF levels through STAT3 could have allowed some CD133^+ ^retinal cells to remain in an arrested progenitor state. Such cells could still respond to LIF signalling but reducing the overall neurosphere numbers as fewer cells have committed to differentiation? After pre-exposure to LIF, cell types within neurospheres generated were not different from those neurospheres generated in FGF/N2 Therefore LIF exposure did not detectably alter cell fate or potential. Neurospheres contained neurons, glial and photoreceptor cells. Although evidence suggests that LIF enhances self-renewal by driving a more glial phenotype in differentiated cells [30,77], we did not observe this in our cultures as relative numbers of GFAP^+ ^cells were not increased.

## Conclusion

These results provide more evidence for the inherent regenerative ability of the adult human retina. Through purification of a CD133^+ ^cell population we have demonstrated that the adult human retina possess cells which can proliferate and whose phenotypic profile after LIF exposure is suggestive of progenitor cell status. The similarity of CD133^+ ^retinal cells with other progenitor populations suggests common themes in the behaviour of progenitor cells from different sources. Emerging evidence (paper in preparation) from human retinal neural progenitor cells suggests that they respond to other members of this cytokine family (which includes LIF, IL-6, CNTF and others). LIF and IL-6 share a common signalling mechanism activating gp130 [77-79]. This makes the study of the transcriptional targets of LIF/STAT3 an important area of future study in CD133^+ ^purified adult human retinal progenitors and suggests a role for LIF in expansion of these cells. However more understanding is needed in the control of what maintains the regenerative ability of cells within the retina and the signals that decide which cells proliferate and which differentiate. Several signalling pathways have been suggested including the Oct-4, Wnt/b-catenin, Notch, BMP (bone morphogenic protein) [80,81] and sonic hedgehog [7]. Further work into this area which, will include in depth analysis of microarray data generated for CD133^+ ^retinal cells (GEO GSE14733) corroborated by quantitative PCR analysis, will enable us to understand how disease modifying treatments can be developed to target these progenitor cells and possible ex-vivo expansion of retinal progenitor cells for transplantation within the future [43,44,54,79-81]. We will assess gene expression within the CD133^+ ^population over time when exposed to mitogens (including BMPR, noggin, sonic hedgehog and jagged-1). The data produced will generate further understanding of the control of these cells within the neural retina.

## Competing interests

The authors declare that they have no competing interests.

## Authors' contributions

DAC carried out, participated in the design and study of the experimental procedures and drafted the manuscript. EJM and ADD participated in the design of the study and helped to draft the manuscript. All authors read and approved the final manuscript.

## Pre-publication history

The pre-publication history for this paper can be accessed here:



## Supplementary Material

Additional File 1 **flow cytometric analysis Of CD133^+ ^retinal cells**. The Fluorescent histograms depicted show the relative expression of stem/progenitor cell markers. Grey filled peaks represent isotype mAb fluorescence for each specific mAb investigated. Numbers within peaks refer to mean fluorescent intensity (MFI) and numbers above bars refer to percentage cell expression. Click here for file
