# Supplementary figures and images for: CD133+ adult human retinal cells remain undifferentiated in Leukaemia Inhibitory Factor (LIF)
Source: BMC Ophthalmol. 2009 Feb 23;9:1. doi: 10.1186/1471-2415-9-1 (PMC2649894; doi:10.1186/1471-2415-9-1)

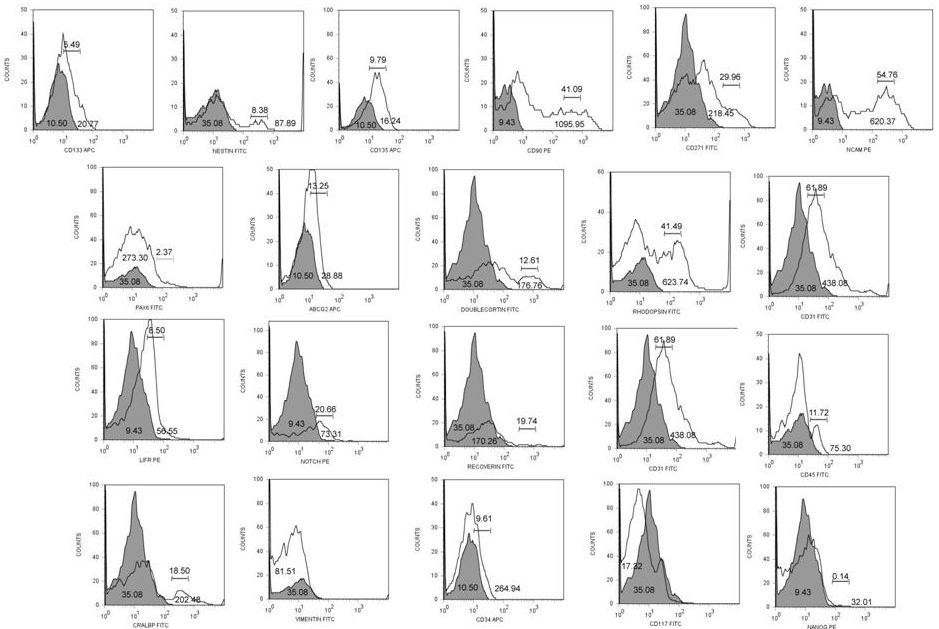

Supplement: Additional File 1 — flow cytometric analysis Of CD133+ retinal cells. The Fluorescent histograms depicted show the relative expression of stem/progenitor cell markers. Grey filled peaks represent isotype mAb fluorescence for each specific mAb investigated. Numbers within peaks refer to mean fluorescent intensity (MFI) and numbers above bars refer to percentage cell expression. [file 1471-2415-9-1-S1.jpeg]
